# Supplementary material for: Machine Learning Accelerates Crystallization for Structure Determination
Source: Angew Chem Int Ed Engl. 2026 May 4;65(25):e1218503. doi: 10.1002/anie.1218503 (PMC13266958; doi:10.1002/anie.1218503)

checkCIF/PLATON report

Structure factors have been supplied for datablock(s) lcz100

THIS REPORT IS FOR GUIDANCE ONLY. IF USED AS PART OF A REVIEW PROCEDURE FOR PUBLICATION, IT SHOULD NOT REPLACE THE EXPERTISE OF AN EXPERIENCED CRYSTALLOGRAPHIC REFEREE.

No syntax errors found. CIF dictionary Interpreting this report

Datablock: lcz100

Bond precision:	C-C = 0.0072 A		Wavelength=1.54184
Cell:	a=27.1484(2)	b=12.8688(1)	c=28.3804(2)
	alpha=90	beta=97.333(1)	gamma=90
Temperature:	100 K		

	Calculated	Reported
Volume	9834.09(13)	9834.09(13)
Space group	C 2/c	C 1 2/c 1
Hall group	-C 2yc	-C 2yc
Moiety formula	C15 H3 Ag3 F18 N6, C12 H10	C15 H3 Ag3 F18 N6, C12 H10
Sum formula	C27 H13 Ag3 F18 N6	C27 H13 Ag3 F18 N6
Mr	1087.04	1087.04
Dx,g cm-3	2.203	2.203
Z	12	12
Mu (mm-1)	15.502	15.502
F000	6240.0	6240.0
F000'	6269.97	
h,k,lmax	34,16,35	34,15,35
Nref	10514	10111
Tmin,Tmax	0.113,0.045	0.305,1.000
Tmin'	0.018	

Correction method= # Reported T Limits: Tmin=0.305 Tmax=1.000
AbsCorr = MULTI-SCAN

Data completeness= 0.962 Theta(max)= 77.923

R(reflections)= 0.0382(9634) wR2(reflections)= 0.1054(10111)

S = 1.081 Npar= 731

The following ALERTS were generated. Each ALERT has the format
test-name_ALERT_alert-type_alert-level.
Click on the hyperlinks for more details of the test.

Alert level C

```
PLAT213_ALERT_2_C Atom F013           has ADP max/min Ratio .....         4.0 prolat
PLAT213_ALERT_2_C Atom F012           has ADP max/min Ratio .....         3.5 prolat
PLAT250_ALERT_2_C Large U3/U1 Ratio for <U(i,j)> Tensor(Resd    1)         3.4 Note
PLAT250_ALERT_2_C Large U3/U1 Ratio for <U(i,j)> Tensor(Resd    3)         2.7 Note
PLAT250_ALERT_2_C Large U3/U1 Ratio for <U(i,j)> Tensor(Resd    4)         2.2 Note
PLAT906_ALERT_3_C Large K Value in the Analysis of Variance ......      2.109 Check
PLAT911_ALERT_3_C Missing FCF Refl Between Thmin & STh/L=    0.600         26 Report
                1  1  0,  -1  1  1,  -2  0  2,   4  0  2,  -5  7  3,  29  5  3,
                2  0  4,   6  0  4,  -9  7  5, -32  0  6,  -9  7  6,   4  0  6,
                6  0  6,   4  0  8,   6  0  8,  30  0  8,  17 11  9,  17 11 10,
               17 11 11,   4  0 12,  17 11 12,  17 11 13,  15 11 15, -30  0 16,
                0  2 17,  14  0 28,
PLAT934_ALERT_3_C Number of (Iobs-Icalc)/Sigma(W) > 10 Outliers ..          1 Check
               12  6 26,
PLAT977_ALERT_2_C Check Negative Difference Density on H01F      .      -0.32 eA-3
```

Alert level G

```
PLAT083_ALERT_2_G SHELXL Second Parameter in WGHT  Unusually Large      53.57 Why ?
PLAT142_ALERT_4_G s.u. on b - Axis Small or Missing .............    0.00010 Ang.
PLAT143_ALERT_4_G s.u. on c - Axis Small or Missing .............    0.00020 Ang.
PLAT242_ALERT_2_G Low    'MainMol' Ueq as Compared to Neighbors of       C01M Check
PLAT242_ALERT_2_G Low    'MainMol' Ueq as Compared to Neighbors of       C021 Check
PLAT242_ALERT_2_G Low    'MainMol' Ueq as Compared to Neighbors of       C025 Check
PLAT242_ALERT_2_G Low    'MainMol' Ueq as Compared to Neighbors of       C01K Check
PLAT242_ALERT_2_G Low    'MainMol' Ueq as Compared to Neighbors of       C01O Check
PLAT242_ALERT_2_G Low    'MainMol' Ueq as Compared to Neighbors of       C01Q Check
PLAT242_ALERT_2_G Low    'MainMol' Ueq as Compared to Neighbors of       C01S Check
PLAT242_ALERT_2_G Low    'MainMol' Ueq as Compared to Neighbors of       C023 Check
PLAT242_ALERT_2_G Low    'MainMol' Ueq as Compared to Neighbors of       C024 Check
PLAT434_ALERT_2_G Short Inter HL..HL Contact F00B     ..F00Z     .       2.80 Ang.
                                               x,1-y,1/2+z  =      6_566 Check
PLAT434_ALERT_2_G Short Inter HL..HL Contact F00E     ..F012     .       2.75 Ang.
                                          3/2-x,1/2+y,3/2-z  =      4_656 Check
PLAT434_ALERT_2_G Short Inter HL..HL Contact F00S     ..F014     .       2.83 Ang.
                                                  x,1+y,z  =      1_565 Check
PLAT720_ALERT_4_G Number of Unusual/Non-Standard Labels .........        102 Note
              Ag01    Ag02    Ag03    Ag04    Ag05    F006    F007    F008
              F009    F00A    F00B    F00C    N00D    F00E    F00F    F00G
              F00H    N00I    N00J    N00K    F00L    F00M    N00N    F00O
              F00P    N00Q    N00R    F00S    N00T    F00U    F00V    N00W
              F00X    F00Y    F00Z    F010    F011    F012    F013    F014
```

```
             C015    C016    C017    C018    C019    C01A    C01B    C01C
             C01D    H01D    C01E    H01E    C01F    H01F    C01G    H01G
             F01H    C01I    C01J    C01K    C01L    H01L    C01M    C01N
             C01O    C01P    H01P    C01Q    C01R    H01R    C01S    C01T
             H01T    C01U    C01V    H01V    C01W    H01W    C01X    H01X
             C01Y    H01Y    C01Z    H01Z    C020    H020    C021    C022
             H022    C023    C024    C025    C026    H026    C027    H027
             C028    H028    C029    H029    C02A    H02A
PLAT883_ALERT_1_G Absent Datum for _atom_sites_solution_primary ..     Please Do !
PLAT910_ALERT_3_G Missing FCF Reflection(s) Below Theta(Min)[Deg]=       3.28 Note
               0  0  2,
PLAT912_ALERT_4_G Missing # of FCF Reflections Above STh/L=  0.600        334 Note
PLAT913_ALERT_3_G Missing # of Very Strong Reflections in FCF ....          3 Note
               4  0  2,   2  0  4,   6  0  6,
PLAT941_ALERT_3_G Average HKL Measurement Multiplicity ..........         3.0 Low
PLAT969_ALERT_5_G The 'Henn et al.' R-Factor-gap value ..........       3.691 Note
             Predicted wR2: Based on SigI**2  2.86 or SHELX Weight  9.75
PLAT978_ALERT_2_G Number C-C Bonds with Positive Residual Density.          0 Info
```

---

   0 **ALERT level A** = Most likely a serious problem - resolve or explain
   0 **ALERT level B** = A potentially serious problem, consider carefully
   9 **ALERT level C** = Check. Ensure it is not caused by an omission or oversight
  23 **ALERT level G** = General information/check it is not something unexpected

```
   1 ALERT type 1 CIF construction/syntax error, inconsistent or missing data
  20 ALERT type 2 Indicator that the structure model may be wrong or deficient
   6 ALERT type 3 Indicator that the structure quality may be low
   4 ALERT type 4 Improvement, methodology, query or suggestion
   1 ALERT type 5 Informative message, check
```

Validation response form

Please find below a validation response form (VRF) that can be filled in and pasted into your CIF.

```
# start Validation Reply Form
_vrf_PLAT213_lcz100
;
PROBLEM: Atom F013           has ADP max/min Ratio .....        4.0 prolat
RESPONSE: ...
;
_vrf_PLAT250_lcz100
;
PROBLEM: Large U3/U1 Ratio for <U(i,j)> Tensor(Resd    1)        3.4 Note
RESPONSE: ...
;
_vrf_PLAT906_lcz100
;
PROBLEM: Large K Value in the Analysis of Variance ......      2.109 Check
RESPONSE: ...
;
_vrf_PLAT911_lcz100
;
PROBLEM: Missing FCF Refl Between Thmin & STh/L=    0.600         26 Report
RESPONSE: ...
;
```

```
_vrf_PLAT934_lcz100
;
PROBLEM: Number of (Iobs-Icalc)/Sigma(W) > 10 Outliers ..          1 Check
RESPONSE: ...
;
_vrf_PLAT977_lcz100
;
PROBLEM: Check Negative Difference Density on H01F      .      -0.32 eA-3
RESPONSE: ...
;
# end Validation Reply Form
```

It is advisable to attempt to resolve as many as possible of the alerts in all categories. Often the minor alerts point to easily fixed oversights, errors and omissions in your CIF or refinement strategy, so attention to these fine details can be worthwhile. It is up to the individual to critically assess their own results and, if necessary, seek expert advice.

PLATON version of 17/09/2025; check.def file version of 17/09/2025

---

duplicate check

No duplication found

Datablock lcz100 - ellipsoid plot

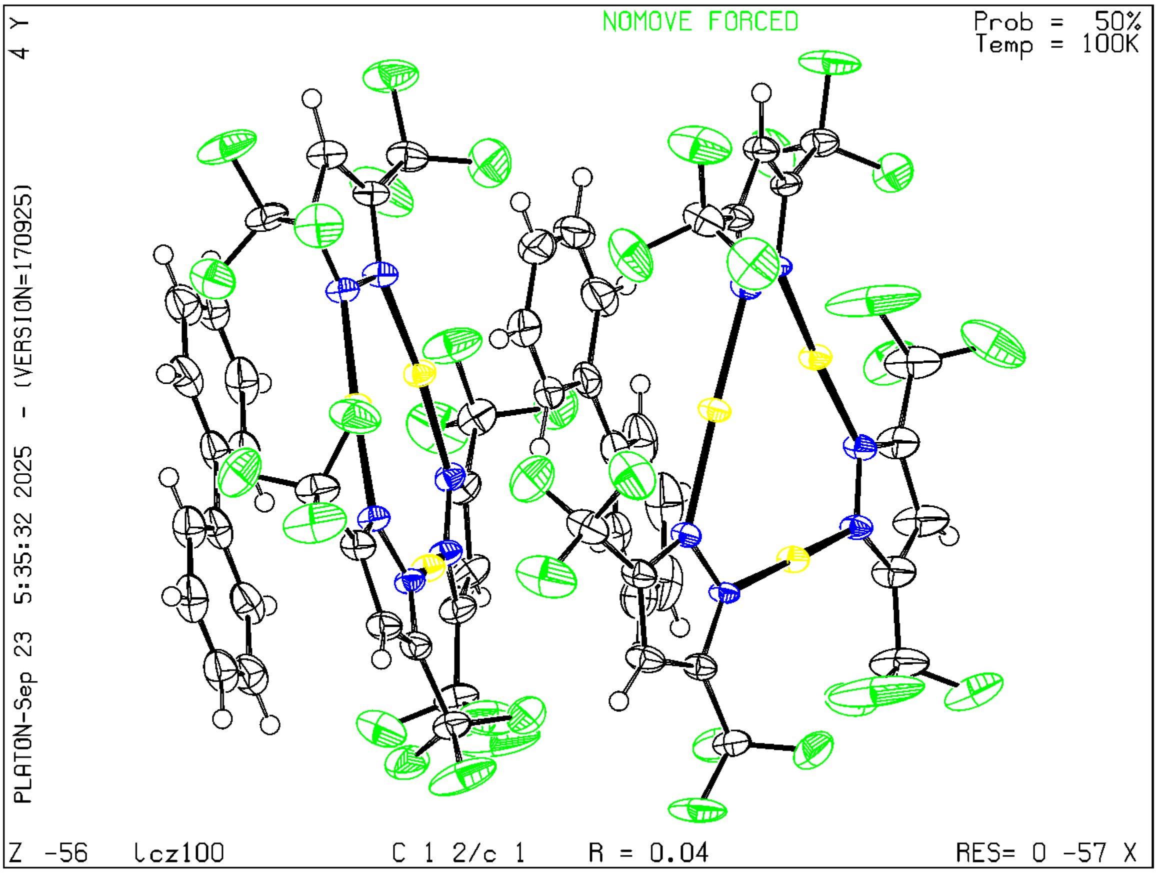

Supplement: Supplementary file 2 — Supporting File 2: anie72462‐sup‐0002‐DataFile.zip. [file ANIE-65-e1218503-s002.zip › supplementary_materials_crystallographic_data submit.zip!/cif_and_checkcif(1-114)/Ag3Pz3_100/Ag3Pz3_100_checkcif.pdf]
